# Multiple Stressor Differential Tolerances: Possible Implications at the Population Level

**DOI:** 10.1371/journal.pone.0151847

**Published:** 2016-03-18

**Authors:** Cátia Venâncio, Rui Ribeiro, Amadeu Soares, Isabel Lopes

**Affiliations:** 1 Department of Biology & CESAM, University of Aveiro, Campus de Santiago, 3810–193 Aveiro, Portugal; 2 CFE–Centre for Functional Ecology, DepartmentofLifeSciences, Universityof Coimbra, Calçada Martim de Freitas, 3000–456 Coimbra, Portugal; Stockholm University, SWEDEN

## Abstract

The probability of the most sensitive genotypes being eliminated from a population due to a contaminant pulse–genetic erosion–is negatively associated to the within-genotype variation. A sensitive genotype with a small phenotypic variation would be more prone to be lost–a critically sensitive genotype. Furthermore, natural populations inhabiting contaminated sites are usually exposed to several pollutants. Such co- or sequential exposure can have severe effects if at least some tolerant clonal lineages surviving one contaminant are sensitive to the others. Such an inverse relationship coupled with a low within-genotype variation potentially enhances genetic erosion. Accordingly, this study evaluated co-tolerance and the occurrence of clonal lineages critically sensitive to 48-hours lethal exposures of copper, zinc, cobalt, and chromium among eight clonal lineages of the cladocerans *Daphnia longispina*. Median lethal concentrations (LC_50_) of each metal were found to have the potential to provoke genetic erosion. Pairwise comparisons of LC_50_, from the eight clonal lineages, revealed neither negative nor positive correlations (r ≤ |0.56|; p ≥ 0.18), but inversely sensitive clonal lineages were found for all pairs of metals. Therefore, besides having the potential to eliminate critically sensitive clonal lineages in a first intermediately lethal pulse, all tested metals may provoke further losses of clonal lineages in an already genetically eroded population.

## Introduction

When conducting ecological risk assessments of contaminated sites, more and new ecological relevant aspects should be introduced to increase the accuracy of risks characterisation, and, thus, the protection of ecosystems. One of such aspects is that natural populations are genetically variable, i.e. they hold a number of different genotypes that will respond differently to chemical contamination. Actually, if the intensity of a stressor is strong enough, then it may lead to the disappearance of the most sensitive genotypes from the initial population, causing genetic erosion. Since the genetic diversity is the basis for adaptation of populations through natural selection, its reduction may compromise the resilience and adaptation of populations to subsequent environmental change, increasing their extinction risk [1–3]. Low values of among- and/or within-genotype variation (i.e. the expression of different phenotypes from the same genotype-phenotypic plasticity), in the tolerance to a contaminant are of particular concern because small differences in the stress intensity will more easily determine the disappearance (i) of the entire set of genotypes, when among-genotype (i.e. genetically determined) variation is low and/or (ii) of each of the sensitive genotypes, when within-genotype variation (i.e. the expression of different phenotypes from the same genotype-phenotypic plasticity) is low. In the literature, several works have already reported the loss of genetic diversity in natural populations exposed to chemical contamination [3–9]. As an example, Lopes *et al*. [10] and Agra *et al*.[11] observed that populations of the cladocerans *Daphnia longispina* historically exposed to metal contamination were less genetically diverse comparatively to reference populations inhabiting the same aquatic system. In addition, these authors also reported that the most sensitive clonal lineages to metal contamination were not present in the historically metal-exposed population of *D*. *longispina*.

A population genetically eroded due to exposure to a certain chemical will most probably be composed by tolerant genotypes to that specific chemical [3], [10], [11]. Whether these genotypes are tolerant or sensitive to other chemicals may determine the survival and persistence of this population in a situation of subsequent inputs of different chemicals. If the remaining metal tolerant genotypes are more sensitive to other type of stressors, than this population will be at a higher risk of being wiped-out. Actually, the association between tolerance to more than one toxicant, namely for several metals, has already been reported by several works (e.g. [12], [13], [14]). For example, Lopes *et al*. [14] observed an association between tolerance to lethal levels of zinc and copper for different genotypes of *D*. *longispina*. Making a parallel to the hypothetical scenarios proposed by Vinebrooke *et al*. [15] on the role of species co-tolerance on the effects of several stressors on biodiversity and ecosystem functioning, populations presenting variation in genetically determined tolerance face two alternative interactions. First, the existence of a strong positive correlation between the tolerances to the different contaminants will guarantee that remaining genotypes after the first exposure will also tolerate a later input of a different chemical. Second, the existence of some genotypes tolerant to the first chemical but sensitive to the second–inversely sensitive genotypes–will result in further genetic erosion: a second round of genotype loss. This latter alternative–the multiple stressors differential tolerance (working) hypothesis [16]–will be disastrous if a strong negative correlation between the tolerance to the two chemicals exists in the population: all the remaining genotypes, after the first exposure, risk to be eliminated by the later contaminant [16].

In the present study, co-tolerance to lethal levels of contamination in eight clonal lineages of the cladocerans *D*. *longispina* was evaluated for all possible pairs of the metals copper, zinc, cobalt, and chromium. If positive correlations are found, then the tolerant clonal lineages to a given metal will be tolerant to other metals. However, if negative linkages between tolerance to several metals occur, then at least some clonal lineages tolerant to a specific metal may disappear in the subsequent presence of other metals. Subsequent pulses of different metals, at partially lethal concentrations of these metals, would, under this scenario, severely increase the number of lost clonal lineages–the multiple stressors differential tolerance hypothesis [16].

## Materials and Methods

### Test organisms

Eight clonal lineages of *Daphnia longispina* O. F. Müller known to exhibit different genetically determined tolerance to lethal levels of acid mine drainage and copper [10], [17]were selected to perform this study: E84, E89, N37, N31, N22, N116, N91, and E99, in increasing order of sensitivity to lethal levels of copper. *Daphnia* is a genus commonly used in ecotoxicological studies because it reproduces by cyclic parthenogenesis, which allows maintaining in the laboratory exactly the same clone for several generations. In addition, these clones of *D*. *longispina* have long been studied, being their allozyme and microsatellite haplotypes previously characterised by Martins *et al*.[18]and Silva *et al*. [19], respectively. The eight clonal lineages were isolated from artificial reservoirs of the downstream portion of the Chança River basin (Southeast Portugal; 37° 37’ N, 7° 30’ W)[10]. Cultures of each clonal lineage were maintained in laboratory, for more than 350 generations, under controlled conditions of temperature (19 to 21°C) and photoperiod (14:10 h L:D) in ASTM hardwater (American Society for Testing and Materials) [20] with vitamins and the organic additive Marinure 25 (an extract from the algae *Ascophyllum nodosum*; Pann Britannica Industries Ltd., Waltham Abbey, UK) [21]. Prior to assays, cultures of all cloned lineages were maintained by changing the medium every other day and organisms were fed daily with the green algae *Pseudokirchneriella subcapitata*(Korshikov) F. Hindák (formerly known as *Selenastrum capricornutum*) (1.5 x 10^5^ cells/mL/d). All clonal lineages were maintained by asexual reproduction, being neonates selected to maintain laboratory cultures and carry out the toxicity assays those from third, fourth, or fifth broods.

### Tested chemicals

Four metals (copper, zinc, cobalt, and chromium) were tested. Metal stock solutions of 80 mg Cu/L, 1.0 g Zn/L, 1.0 g Co/L, and 1.0 g Cr/L were prepared with copper sulphate (CuSO_4_), zinc sulphate (ZnSO_4_.7H_2_O), cobalt chloride (CoCl_2_.6H_2_O), and potassium dichromate (K_2_Cr_2_O_7_) (all purchased from Sigma-Aldrich, St. Louis, USA) dissolved in nanopure water (Milli-Q Academic System; Millipore, MA, USA).

### Experimental Design

Lethal assays with each clonal lineage exposed to each metal were carried out according to OECD (2004) guidelines [22]. After performing preliminary assays, the gradients of definitive concentrations were set for each metal. Each clonal lineage was exposed to a control (ASTM hardwater) and to five concentrations of each metal, with a dilution factor of 1.4 times. The range of concentrations varied among clonal lineages, depending on their sensitivity to each metal (Table 1).

**Table 1:** Description of the concentration ranges tested for each clonal lineage. The dilution factor used for all serial dilutions was 1.4x.

	Cu	Zn	Co	Cr
**E84**	13.0–50.0	612.2–2352	2500–9604	416.6–1372
**E89**	25.3–97.1	570.0–2190	2142–8232	300.0–1152
**37**	51.1–196	571.4–2195	1500–5762	1000–3841
**N31**	55.5–115	845.2–3247	2520–9681	120–461
**N22**	90.0–346	840.0–3227	3500–13,445	600.0–2305
**N116**	118–243	700.0–2689	1260–4840	571.4–2305
**N91**	145–669	752.9–2892	2520–9681	385.7–1482
**E99**	150–311	768.3–2951	1764–6777	385.7–1482

Dilutions of the stock solutions were made with ASTM hardwater medium. Metal analyses were considered unnecessary because the objective of this work was solely the comparison of among-lineages relative sensitivities to the tested chemicals, and not to determine absolute self-standing effective concentrations of each chemical to each cloned lineage.

All assays were carried out for 48 hours at 19 to 21°C and at a 16h:8h light:dark photoperiod, with neither food addition nor medium renewal. Five 6 to 24-hour-old neonates were placed in 50ml vessels containing 30 ml of test solution with four replicates per clone and concentration. Mortality at 24 and 48 hours was quantified considering an organism as dead if immobile during 15 s after gentle prodding. Conductivity (Wissenschaftlich Technische Werkstätten-WTW conductivity440i, Weilheim, Germany), pH (WTW pH330i) and dissolved oxygen (WTW OXI 330i) were registered at the beginning and end of each assay.

Prior to directly testing the multiple stressors differential tolerance (working) hypothesis, by exposing assemblages of different genotypes to simultaneous and/or sequential inputs of different contaminants, more knowledge on the occurrence of inversely sensitive genotypes is needed. Such data will allow predicting for which mixtures the working hypothesis is expected to be confirmed and this was what the present study aimed at.

### Data analysis

Median, lower and upper quartile lethal concentrations (LC_50_, LC_25_, and LC_75_, respectively) were calculated, for each clonal lineage, using the software Priprobit [23]. For each metal, phenotypic variance (V_P_) was computed by adding the environmental variance (V_E_) and the genetic variance (V_G_), V_E_ being determined with approximate estimates of standard deviation values from the models fitted to mortality versus concentration and approximate values of broad sense heritability for each metal being determined as the quotient of V_G_ over V_P_ [24]. For comparative purposes, the among-genotype variation was quantified with the coefficients of variation (CV) of the median lethal concentrations. The within-genotype variation was quantified with the relative spread of sensitivity to each metal of each clonal lineage: the difference between the lower (LC_25,48h_) and upper (LC_75,48h_) quartiles divided by the median (LC_50,48h_), in percentage.

For each metal, a clonal lineage was categorised as critically sensitive if its LC_75,48h_ was close to or below the average of the set of LC_50,48h_ for the eight clonal lineages. For each pair of metals, inversely sensitive clonal lineages, being of special concern within this study’s rationale, were those critically sensitive to one of the metals but not to the other [16]. Therefore, safely co-tolerant clonal lineages were those neither critically co-sensitive nor inversely sensitive. Pearson’s correlation coefficients between LC_50,24h_ and LC_50,48h_ values for each pair of metals were determined using the program Statistica for Windows 4.3 (StatSoft, Aurora, CO, USA).

## Results

**Fig 1:**
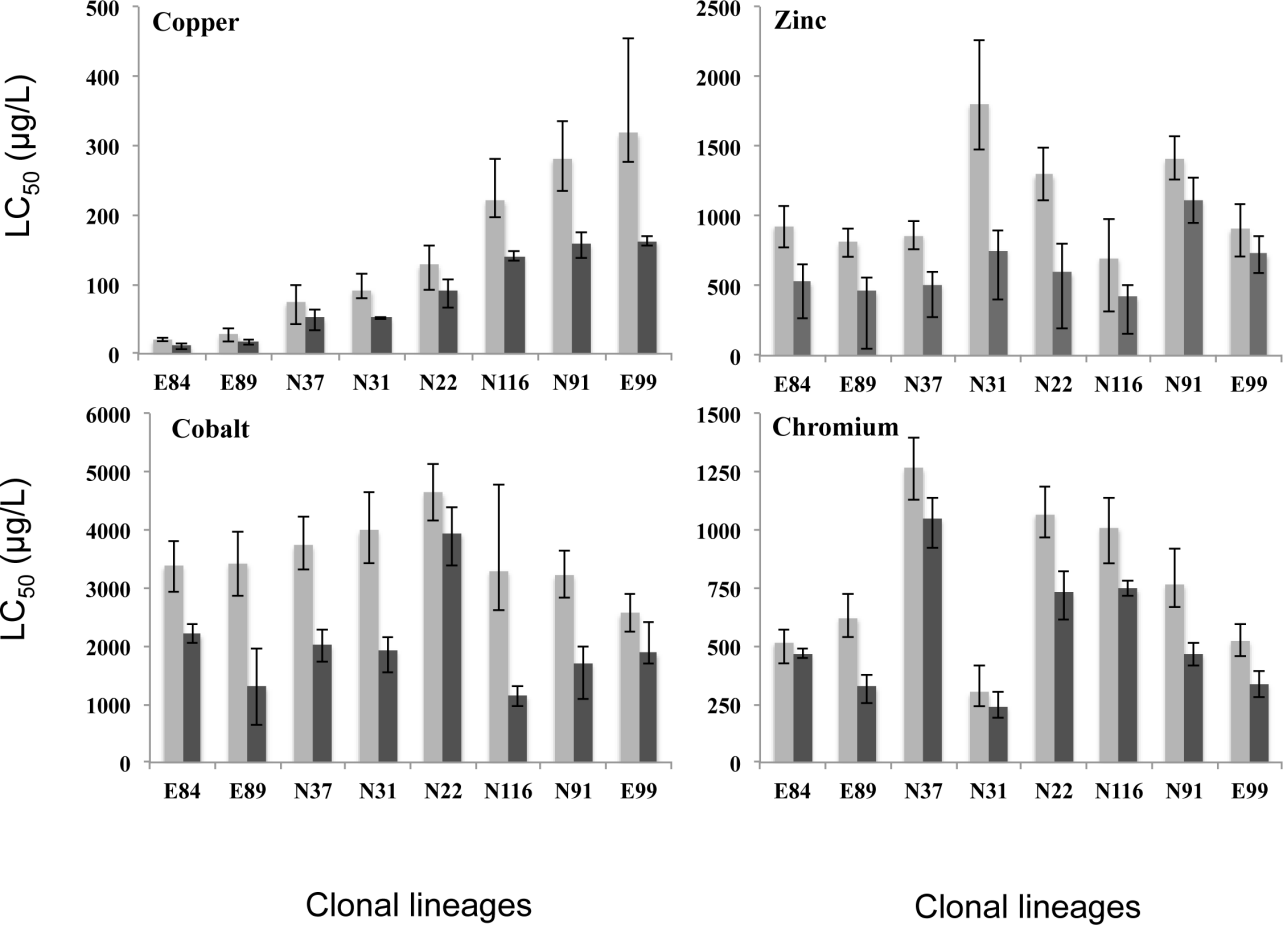
Values of the medial lethal concentration (LC_50_), with the respective 95% confidence limits (error bars), computed after 24 (light bars) and 48h (dark bars) of exposure, for the eight clonal lineages of *Daphnia longispina* exposed to the four tested metals (copper, zinc, cobalt and chromium).

Dissolved oxygen, pH and conductivity presented very small variations during the assays, being always above 7.2 mg/L, close to 8 and close to 540 μS/cm, respectively. The largest among-genotype variation in the tolerance to lethal levels after a 48-hour exposure was found for copper(CV of 72%); the most sensitive clonal lineage (E84: LC_50,48h_ = 12 μg/L) being14 times more sensitive than the most tolerant one (E99: LC_50,48h_ = 163 μg/L), while for zinc, cobalt and chromium the variability was lower (CV values of 35, 43 and 50%, respectively) (Fig 1). Within-genotype variation, measured as the mean of relative spreads of sensitivity (observed range inside brackets), was 40 (6 to 78%), 56 (43 to 69%), 44 (10 to 88%), and 46% (9 to 110%), for copper, zinc, cobalt, and chromium respectively; with three, two, two, and four out of eight clonal lineages being critically sensitive to each of these metals, respectively. One clonal lineage (E89) was found to be critically sensitive to the four metals. Not only was this clonal lineage different from all others, but also all clonal lineages presented patterns of sensitivity to lethal levels of metals that differed from each other. Approximate estimates of broad-sense heritability for each metal were 74, 18, 37, and 51%, respectively.

**Fig 2:**
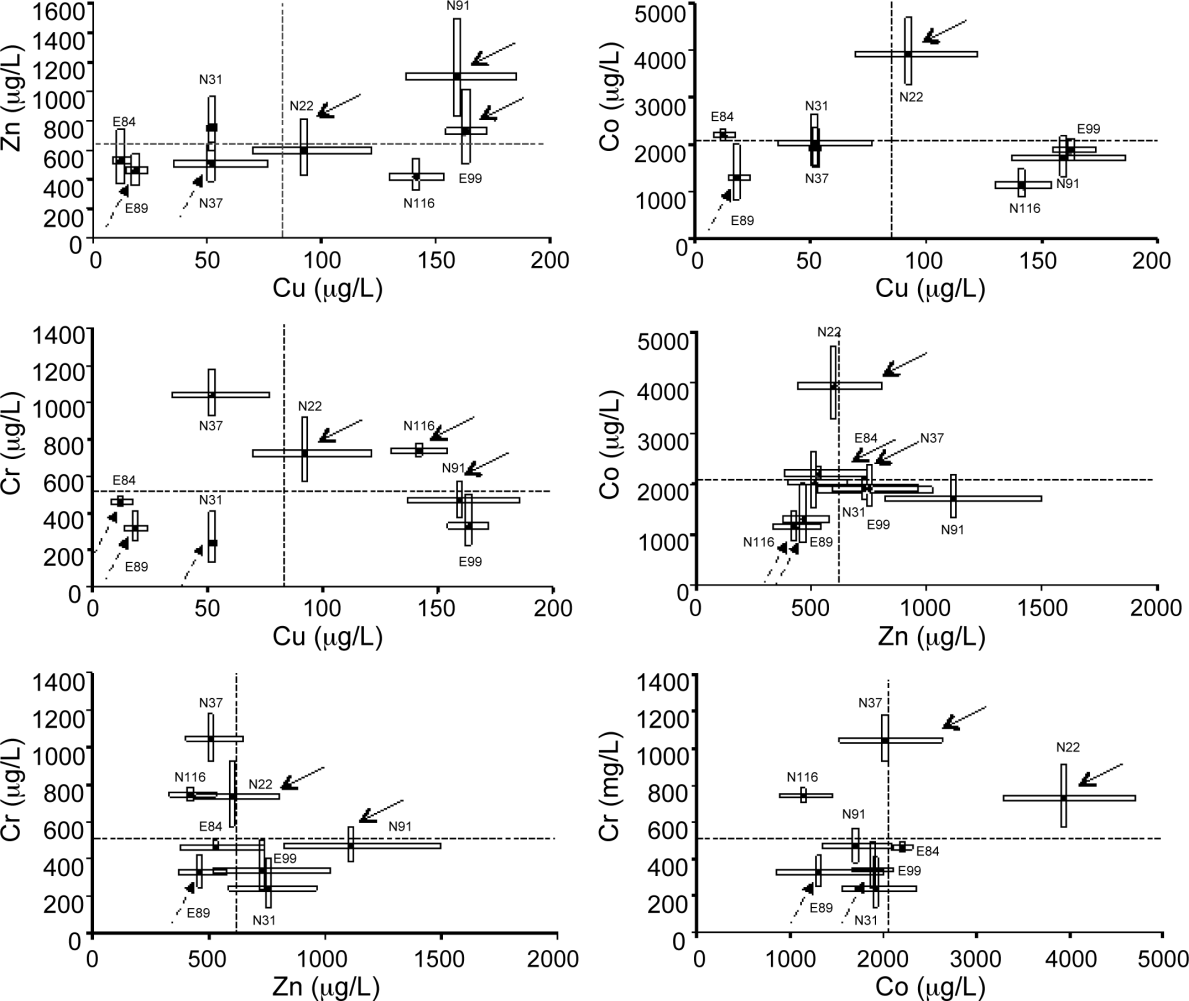
Boxplots (lower quartile, median and upper quartile, i.e. LC_25_, LC_50_ and LC_75_, respectively) for the eight clonal lineages of *D*. *longispina* and for each pair of the four tested metals (copper, zinc, cobalt, and chromium). Black circles indicate LC_50_ values and boxes represent the LC_25_ and LC_75_ values of each metal for each clonal lineage, after a 48-h exposure. Dashed lines indicate the mean of the set of LC_50,48h_ for the eight clonal lineages. Solid arrows indicate the safely co-resistant clonal lineages and dashed arrows indicate the critically co-sensitive for each pair of metals.

Significant correlations between median lethal concentrations for each pair of the tested metals were found neither at 24 nor at 48 hours (r ≤ |0.56|; p ≥ 0.18). The worst-case scenarios for paired exposures were Cu|Co with six inversely sensitive clonal lineages, Zn|Cr with five, and Co|Cr with four (Fig 2). For the former pair, only one lineage was safely co-tolerant (N22), which was also safely co-tolerant in the other five combinations of the four tested metals.

## Discussion

The genetic variation in the tolerance to copper of the eight clonal lineages of *D*. *longispina* was not expected to be, by any means, representative of natural populations, because these lineages were selected aiming at maximising the CV parameter. Therefore, the CV of 72% for the LC_50,48h_ values probably overestimates what would be expectable in natural populations. Nevertheless, a high among-genotype variation was also found for the other metals (CV values of 35 to 50%), which is in accordance with other studies on both laboratory reared and field collected clonal lineages of daphnids. For instance, CV of 53 and 52% for Cu and Zn, respectively, were determined for *D*. *longispina* populations acclimatised to laboratorial conditions [25] and CV values ranging from 39 to 61% for *D*. *longispina* exposed to 250 μg/L Cu [26]. A low among-genotype variation in sensitivity to a contaminant would indicate a small difference between partially lethal and fully lethal concentrations, at the population level, and, thus, a small increase in environmental concentrations would more easily determine the elimination of all genotypes. The opposite scenario, with a high intraspecific variability, would be an apparently safer situation, since a small increase in environmental concentrations would potentially eliminate only a few more, if any, genotypes. However, this latter scenario should be the one of most concern because of the higher probability of using, in laboratory toxicity testing, a genotype or even a population, which would not be representative of the species sensitivity. The classical and almost exclusive approach of ignoring and, even worse, minimising intraspecific genetic variation in the sensitivity to contaminants by, for instance, using a single daphnid clone, should, therefore, be increasingly questioned.

Though measures of among-genotype variability are needed to appraise the risk of population extinction, insights on the risk of contaminant-driven genetic erosion by natural selection demand for the evaluation of within-genotype variability. Genotypes with low relative spreads of sensitivity for each metal, such as N31 with copper, N116 with chromium, E84 with cobalt and E99 with copper (6, 9,10 and 10% of relative spread values, respectively) would be easily wiped out by a small increase of contaminant environmental concentrations. If clonal lineages here used were representative of natural reference populations, which cannot be assumed, then zinc and cobalt, with the lowest number of critically sensitive genotypes–two out of eight–were the metals posing the lowest relative risk of genetic erosion. The proportion of critically sensitive clonal lineages, regarding copper and chromium (three and four out of eight, respectively), pointed to a higher risk of genetic erosion by these two metals.

One clonal lineage (E89) was found to be critically sensitive to all tested metals, being always at a high risk and, therefore, the subsequent exposure to intermediately lethal levels of two metals would not change much the risk of its most probable elimination. It is the number of inversely sensitive genotypes that expresses the increased susceptibility to subsequent stressors. Considering the potential for genetic erosion due to an intermediately lethal pulse of each tested metal, it would be expected that 37.5, 25, 25, and 50% of the clonal lineages could be eliminated by copper, zinc, cobalt, and chromium, respectively. However, the loss of genotypes could more than double, attaining 88%, if a pulse of copper was followed or preceded by a pulse of cobalt, both at intermediately lethal concentrations (the mean of the set of LC_50,48h_), assuming that all clonal lineages critically sensitive to each metal would be killed.

These scenarios are even possible when considering the egg banks of *D*. *longispina*, instead of the population resulting from parthenogenic reproduction in natural populations. In a recent publication, Rogalski [27] assessed and compared the hatching rate of ephippia of *D*. *magna* collected from sediments non-contaminated and contaminated with metals. He observed that daphnia were more probable to hatch when originated from more recently deposited sediments and when metal concentrations were lower. In addition, he reported that the percentage of mortality of hatchling was higher in daphnia hatched from more recent metal contaminated sediments. It can be argued that such differences in organism’s fitness, due to exposure to chemical contamination, instead of being only related to distinct genotypes, it can be related to epigenetic processes. In daphnids, the occurrence of DNA methylation has indeed been reported in scientific literature [28], but categorical evidences for transgenerational persistence of such changes are still lacking [28], [29], [30], [31].

Furthermore, Weider *et al*.[32] studied the genetic diversity, by characterizing four polymorphic enzyme loci, of egg banks of the *D*.*galeata-hyalina* complex at different depth sections of sediment cores sampled at a lake located in Germany. They observed a significant shift in the allelic composition within the different depth ephippia banks. For example, they observed a severe decrease in the frequency of allele M at locus *Pgi* with a parallel increase in the frequency of allele F at locus *Pgm*. They found a positive correlation between the changes in the frequency of these two alleles and changes in total phosphorous concentration, associated with events of eutrophication. Accordingly to the results obtained in the present study and those described above, it suggests that genetic erosion provoked by sequential exposure to pulses of different metals may occur both at the dormant population (i.e. ephippia resting in the sediment) and at the effective population dwelling in the pond.

In the literature, both the presence and absence of co-tolerance to metals have been reported for a set of species. Münzinger and Monicelli [33] reported chromium-tolerant individuals of *D*. *magna* to be also tolerance to copper and nickel, for several monitored demographic parameters, comparatively to chromium-sensitive individuals. Several mechanisms have been described as being responsible for tolerance to more than one metal. For example, plasmids are known to confer tolerance to several metals, namely, to mercury, zinc, cadmium, and lead [33], [34].Hall *et al*.[35] observed that the organic material produced by the copper-tolerant alga *Ectocarpus siliculosus* was able to bind copper more rapidly than the organic material produced by non-tolerant cells. Furthermore, they also observed that the organic material produced by the tolerant cells could detoxify cobalt and zinc externally. Other authors reported common uptake mechanisms for divalent cations like Cd^2+^, Zn^2+^, and Co^2+^ [34], [36], [37]. For example, Bariaud and Mestre [38] showed that the modifications of the membrane permeability to cadmium ions also explained the increased tolerance observed for cobalt and zinc in Cd-tolerant cells of *Euglena gracilis*. In the presented study, an association of lethal tolerance to more than one metal was not observed among the studied cloned lineages, suggesting the presence of different mechanisms responsible for the tolerance to each of the four metals (Cu, Zn, Co, and Cr). These results agree with other works, which hypothesised that tolerance to high metal concentrations may be specific [38]. Essential metals may be subjected to regulation by uptake limitation or through specific accumulation strategies [39]. Two main routes are referred in the literature regarding trace metals uptake, the first being the transport mediated by carrier molecules and the second pump channels [40], [41].

Actually, in scientific literature, some authors also reported the inexistence of multiple or co-tolerance among several metals. Von Frenskell-Insam and Hutchinson [42] observed that seeds of the plant *Deschampsia cespitosa* that were tolerant to nickel did not exhibit an increased tolerance to Zn, though seedlings selected for an elevated tolerance to Zn were also tolerant to Ni. Further, Adriaensen *et al*.[43] observed that a copper-tolerant fungus (*Suillus luteus*) did not reveal co-tolerance to zinc: Zn-tolerant isolates were very sensitive to copper and vice-versa. These authors argued that though Cu and Zn are essential metals, this does not necessarily involve similar mechanisms in the detoxification of these metals. Accordingly, other researchers showed that the genetic basis of metal tolerance in some bacteria and higher plants does not involve co-tolerance for the pair Cu|Zn, since Cu tolerance and Zn tolerance may be under the control of different genes [43], [44]. Guo *et al*. [45], after examining the contribution of metallothioneins on metal tolerance of *Arabidopsis*, observed that different types and different conjugations of metallothioneins are responsible for conferring tolerance to different metals in this species. Also, Tilstone and Macnair [46], [47], after studying several tolerant and non-tolerant populations of the plant *Mimulus guttatus* found that tolerance to many metals observed in this plant was due to multiple metal tolerance, which was caused by independent genetic mechanisms for specific metals.

So far, this discussion tackled only subsequent exposures to different chemicals, but similar outputs are expected for co-exposures, unless strong antagonistic effects occur. This logical deduction–the theorem of enhanced genetic erosion by multiple simultaneous stressors–merits to be experimentally considered when evaluating the toxicity of mixtures.
